# Patient perspectives on the therapeutic profile of botulinum neurotoxin type A in cervical dystonia

**DOI:** 10.1007/s00415-020-10217-7

**Published:** 2020-09-16

**Authors:** Cynthia Comella, Joaquim J. Ferreira, Emilie Pain, Marion Azoulai, Savary Om

**Affiliations:** 1 Department of Neurology, Rush University Medical Center, 1725 West Harrison St. Suite 755, Chicago, IL 60612 USA; 2 Instituto de Medicina Molecular, Faculdade de Medicina, Universidade de Lisboa, Lisbon, Portugal; 3 CNS, Campus Neurológico Sénior, Torres Vedras, Portugal; 4 Carenity, Paris, France; 5 Global Medical Affairs, Ipsen Pharma, Boulogne-Billancourt, France

**Keywords:** Cervical dystonia, Patient, Survey, Treatment, Botulinum toxin, Waning of effect

## Abstract

**Background:**

Botulinum neurotoxin type A (BoNT-A) is an effective pharmacological treatment for the management of cervical dystonia (CD) that requires repeated administration at variable intervals. We explored patient perceptions of the impact of CD and the waning of BoNT-A therapeutic effects.

**Methods:**

An internet-based survey was conducted through Carenity, a global online patient community, from May to September 2019. Eligible respondents were adults with CD who had ≥ 2 previous BoNT-A injections.

**Results:**

209 respondents (81% females; mean age of 49.7 years) met the screening criteria. The mean BoNT-A injection frequency was 3.9 injections/year. The mean reported onset of BoNT-A therapeutic effect was 11.7 days and the time to peak effect was 4.5 weeks. Symptom re-emergence between injections was common (88%); the time from injection to symptom re-emergence was 73.6 days (~ 10.5 weeks). Treatment was not reported to completely abolish symptoms, even at peak effect. However, symptom severity was rated (0 = no symptoms; 10 = very strong symptoms) as lowest at the peak of treatment effects (mean scores ~ 3/10), increasing as the effects of treatment start waning (~ 5.5/10) and was strongest one day before the next session (~ 7–8/10). The impact of CD on quality of life followed the same ‘rollercoaster’ pattern.

**Conclusions:**

This survey highlights the burden of CD symptoms, even in patients undergoing regular treatment. Symptom re-emergence is common and has significant impact on daily activities and quality of life. Greater awareness of the therapeutic profile of BoNT-A treatment should lead to better informed therapeutic discussions and planning.

**Electronic supplementary material:**

The online version of this article (10.1007/s00415-020-10217-7) contains supplementary material, which is available to authorized users.

## Introduction

Cervical dystonia (CD) is a chronic neurological syndrome primarily characterised by involuntary contractions of the cervical muscles of the neck, resulting in twisting and repetitive movements, or abnormal postures. The average age of CD onset is around 41 years old [1, 2], and many patients are working with young families when they are diagnosed [3]. Disability with functional impairment, pain and embarrassment with social withdrawal are common and several studies have highlighted the considerable quality of life burdens associated with having CD [4–6].

Chemodenervation with botulinum neurotoxin type A (BoNT-A) is considered first-line treatment for CD [7, 8]. The toxin exerts its therapeutic effects by blocking neuromuscular acetylcholine transmission at the peripheral nerve terminals. However, over time, the pharmacological effects of BoNT-A at the neuromuscular junction start to wane [9] leading to symptom re-emergence towards the end of the treatment interval. In patients with CD, the therapeutic response profile is characterised by a significant reduction in symptoms as early as 1–2 weeks, with peak effects at approximately 4–6 weeks and waning of benefit at approximately 8–16 weeks [10–12]. Most BoNT-A product labels currently recommend waiting at least 12 weeks between injections [13, 14]. However, a recent observational study reported that patients with longer injection intervals were more likely to be satisfied with their symptom control at peak effect and end of cycle than those with shorter injection intervals [15]. Other surveys have reported that a proportion of patients would prefer a shorter injection interval to match their shorter therapeutic response [16].

While it is clear that patient satisfaction with treatment is lower at end of cycle than at peak effect [15, 16], patient perceptions of treatment efficacy over a full treatment cycle and the patient-related triggers for reinjection have not been well studied. The aim of this online survey was to characterize the profile of symptom re-emergence by exploring patient perceptions of the impact of CD symptoms, how they experience the waning of BoNT-A effects and how it impacts their quality of life.

## Methods

### Survey design

This international online survey was conducted between May 15, 2019 to September 16, 2019 and was available in France, Germany, Italy, the United Kingdom and the United States of America. The structure and contents of the survey were designed in collaboration with the authors and Carenity, an international online patient community for people living with chronic disease (Paris, France). The survey was hosted online and included 32 questions (Appendix 1 in ESM). Questions were designed to document and explore sample characteristics (demographics and medical history), current treatment for CD (including BoNT-A), experiences of symptom re-emergence, impact of symptom re-emergence on quality of life and physician–patient communication about symptom re-emergence.

Most questions were multichoice with some allowing input of free text. Severity of symptoms and impact on quality of life at different timepoints were rated on analogue scales, ranging from 0 (no CD symptoms/no impact on quality of life) to 10 (very strong CD symptoms/very strong impact on quality of life). To assess impact on quality of life, we asked respondents to rate their ability to work, to have social interactions, to drive, to perform daily tasks and sleep disturbance. Questions were reviewed by two individuals living with CD and currently receiving BoNT-A treatment for their symptoms (one in the US and one in Europe) and refined to improve ease of understanding and relevance. The survey was based on respondent self-report and was designed to take approximately 20–25 min to complete. However, there was no set time limit for completion. The survey was translated from the original English version to provide validated French, German and Italian versions.

### Recruitment and survey participants

The survey was conducted in compliance with data protection legislation. Clinical Research Ethics Committee or Independent Review Board approval was not required for this exploratory patient satisfaction survey. All respondents provided informed consent to participate. They were made aware that the research was sponsored by a pharmaceutical company interested in the treatment of CD.

People with a diagnosis of CD (self-report) were invited to participate in the survey via the Carenity social media platform for people living with chronic disease. Patient associations (e.g., Dystonia Europe, the Dystonia Medical Research Foundation or AMADYS) also shared the survey with their members via email and/or newsletters and social media. The survey was hosted on the Carenity patient community platform. Eligible respondents were adult (≥ 18 years old) CD patients currently undergoing treatment with BoNT-A (≥ 2 injections). Patients who had stopped BoNT-A treatment in the last 12 months were also eligible to participate.

### Data analysis

Descriptive statistics were used to summarise all survey data collected in this study.

## Results

### Respondent characteristics

A total of 318 respondents completed the online survey. Of these, 209 respondents met the survey screening criteria and were included in the analyses. Table 1 show the key respondent population characteristics. The mean [95% CI] age of respondents was 49.7 [48.4–51.0] years and the majority (81%) were female. In terms of employment, 73% of respondents younger than 65 years old (i.e., working age respondents) were employed (40% full time and 33% part time). The mean [95% CI] time since symptoms was 9.8 [8.2–11.4] years and the time since diagnosis was 6.5 [5.5–7.6] years.

**Table 1:** Respondent characteristics

Characteristic	Respondent Population *N* = 209
Country; *n* (%)	
France	38 (18)
Germany	28 (13)
Italy	12 (6)
United Kingdom	34 (16)
United States of America	97 (47)
Sex; *n* (%)	
Female	169 (81)
Male	40 (19)
Age (years); mean [95% CI]	49.7 [48.4, 51.0]
Age category; *n* (%)	
< 40 years old	33 (16)
41–50 years old	88 (42)
51–60 years old	60 (29)
> 60 years old	28 (13)
Age at CD first symptoms (years); mean [95% CI]	39.6 [38.1, 41.2]
Age at CD diagnosis (years); mean [95% CI]	43. 2 [42.0, 44.4]
Time since CD first symptoms (years); mean [95% CI]	9.8 [8.2, 11.4]
Time category; *n* (%)	
< 2 years	22 (11)
2–5 years	86 (41)
6–10 years	34 (16)
> 10 years	58 (28)
Do not remember	9 (4)
Time since CD diagnosis (years); mean [95% CI]	6.5 [5.5, 7.6]
Time category; *n* (%)	
< 2 years	42 (20)
2–5 years	92 (44)
6–10 years	27 (13)
11–20 years	31 (15)
> 20 years	14 (7)
Do not remember	3 (1)
Employment status; *n* (%)^a^	*N* = 192
Full time	77 (40)
Part time due to CD	57 (30)
Part time (not due to CD)	7 (3)
Do not work due to CD	36 (19)
Do not work (not due to CD)	15 (8)

^a^Respondents aged < 65 years old. *CD* cervical dystonia, *CI* confidence interval

Taken overall, four out of 10 patients were diagnosed ≥ 2 years after experiencing their first symptoms. The mean [95% CI] delay between first symptoms and diagnosis was 3.3 [2.2, 4.4] years.

### Cervical dystonia symptoms

Respondents reported experiencing ≥ 4 symptoms of CD within the past 12 months (mean [95% CI] of 4.4 [4.1–4.6] symptoms); the most commonly reported symptoms were pain (86%) and muscle spasms (71%). Around half of all patients said they had experienced difficulties at work, fatigue or lack of energy, feeling sad or depressed and difficulties falling or staying asleep due to their CD symptoms in the past 12 months. Other reported experienced symptoms and situations are displayed in Table 2.

**Table 2:** Cervical dystonia related symptoms and experiences during past year

	Respondent population *N* = 209
Symptoms experienced in past year; *n* (%)^a^	
Neck pain or other related pain	179 (86)
Muscle spasms	149 (71)
Abnormal positioning of the head/neck	145 (69)
Involuntary movement of head or shoulders	133 (64)
Inability to move the head easily	120 (57)
Tremor	95 (45)
Shoulder elevation	92 (44)
Other^b^	5 (2)
Situations experienced in past year; *n* (%)^c^	
Difficulties at work	113 (54)
Fatigue or lack of energy	111 (53)
Feeling sad or depressed	108 (52)
Difficulties staying asleep	106 (51)
Loss of self-confidence	103 (49)
Feeling not refreshed after an overnight sleep	100 (48)
Walking difficulties or balance problem	95 (45)
Pain not explained by other conditions	87 (42)
Difficulties while eating	81 (39)
Experience of unpleasant sensations	77 (37)
Feeling nervous, worried or frightened	76 (36)
Experience of light-headedness or dizziness	70 (33)
Dystonia affecting vision	60 (29)
Problems with (or less interested in) sexual activities	48 (23)
Flat moods without the normal ‘highs and lows’	45 (22)
Speech problems	45 (22)
Other^d^	4 (2)

^a^Question: During the past 12 months, at the worst time, which of the following symptoms have you experienced as a consequence of your cervical dystonia?

^b^Other symptoms were vision disorders (*n* = 2), walking difficulties (*n* = 1), insomnia (*n* = 1) and psychological disorder (*n* = 1)

^c^Question: During the past 12 months, at the worst time, which of the following situations have you experienced as a consequence of your cervical dystonia?

^d^Other situations were arm weakness (*n* = 1), have to rest between tasks (*n* = 1), difficulties doing household tasks (*n* = 1), difficulties performing re-education at home (*n* = 1)

### Current treatment for cervical dystonia

**Fig. 1:**
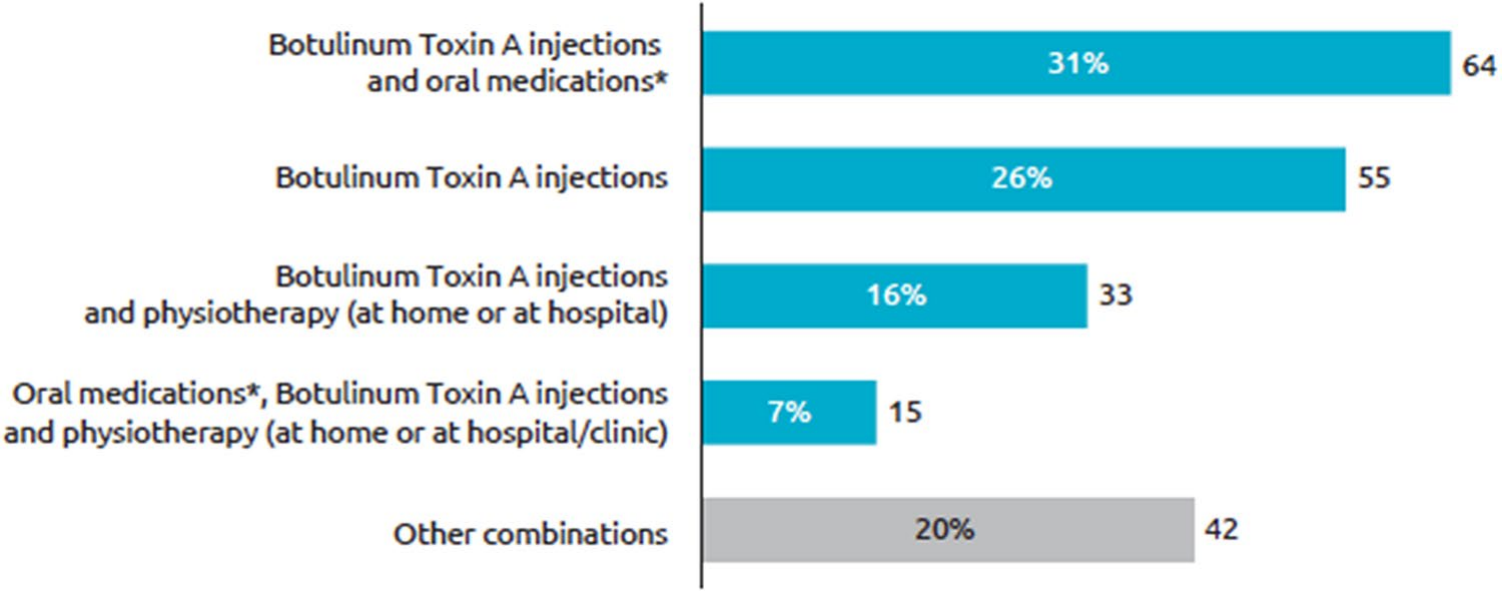
Current therapy for CD symptoms. Question: Which treatments are you currently receiving for your CD? (*N* = 209). *Oral medications such as muscle relaxant or baclofen. *BoNT-A* botulinum neurotoxin type A, *CD* cervical dystonia

As per eligibility criteria, most (98%) respondents were currently receiving BoNT-A therapy (onabotulinumtoxinA 44%, abobotulinumtoxinA 25%, incobotulinumtoxinA 24%, product unknown 7%). The mean [95% CI] duration of BoNT-A therapy was 5.5 [4.6, 6.5] years, and 90% of respondents had received ≥ 4 prior injections. While the majority of respondents said they were promptly treated with BoNT-A following their diagnosis (84% were treated within 2 years), 10% of respondents had a delay of 2–5 years between diagnosis and BoNT-A treatment and 4% had ≥ 6-year delay. Most respondents were treated with ≥ 1 treatment approach (Fig. 1), with almost half (47%) also receiving oral medications for their CD symptoms and a third (33%) having concomitant physiotherapy.

### Botulinum neurotoxin type A injection experiences

The mean injection frequency for CD was 3.9 [3.8, 4.0] injections per year. The majority of respondents said they received three (*n* = 39, 19%), four (*n* = 122, 58%) or five (*n* = 20, 10%) injections per year. Only 6% respondents received ≤ 2 injections and 4% received 6 injections per year. Accordingly, 70% of respondents said they had their last two sessions within 3–4 months of each other, 14% within 4–6 months, while 14% reported having injections with intervals of < 3 months and 2% reported injection intervals of more than 6 months. Most (*n* = 125, 60%) respondents said their injection interval was always the same, and of these, most (80%) said the schedules were well-adapted to their needs. Conversely, of the 28 respondents (13%) who said their injection sessions were based on doctor availability, 71% (*n* = 20) reported that their injections were not adapted to their needs.

**Fig. 2:**
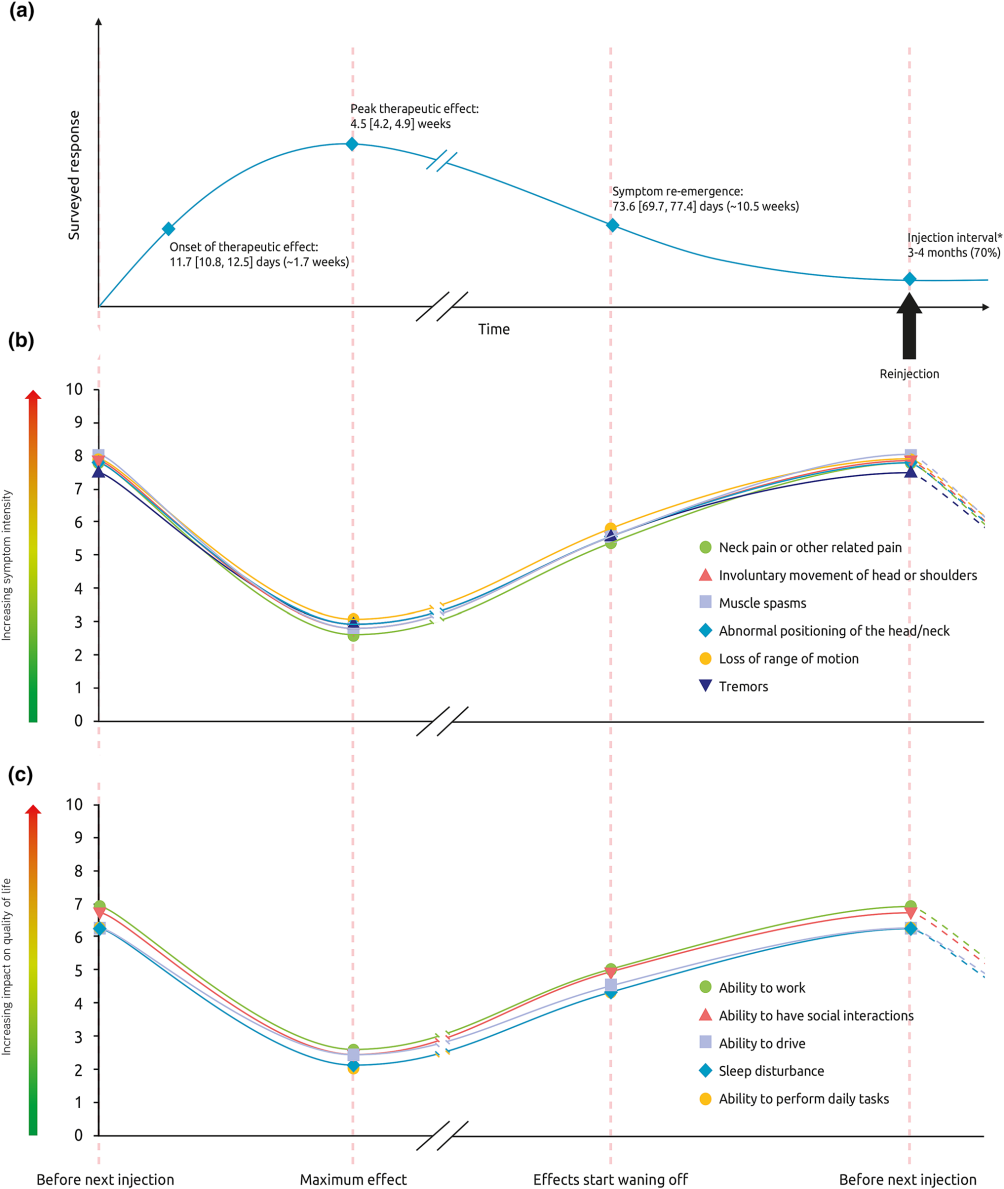
Patient experiences of **a** BoNT-A response **b** CD symptom intensity and **c** impact on quality of life across a single injection cycle. **a** Schematic representing the mean [95% CI] time to onset and peak therapeutic effects and mean time to symptom re-emergence. Respondents were asked: On average, how many days or weeks after your BoNT-A injections do you experience (**1**) The first effect of the treatment on your cervical dystonia (**2**) the maximum effects of the treatment on your CD (in days or weeks). In general, how long after your BoNT-A injections do your pre-existing symptoms begin to reappear? *N* = 209 respondents. *Respondents indicated the time between the last 2 BoNT-A sessions. Figures **b **and** c**: Schematics representing mean symptom intensity (**b**) and impact of CD symptoms on quality of life (**c**) across an injection cycle. Respondents were asked to rate the intensity of symptoms and impact of symptoms on quality of life [scale 0–10] at three different points of treatment: peak effect, waning of effect, just prior to next injection. *N* = 183 respondents whose symptoms reappear between two sessions of injections. *BoNT-A* botulinum neurotoxin type A, *CD* cervical dystonia

When asked to describe the onset of therapeutic effect, 28% of respondents said they noticed effects within 9 days, 46% said they noticed effects within 10–14 days and 16% said it took > 15 days to notice the first effects of BoNT-A treatment on their cervical dystonia. Overall, the mean [95% CI] onset was 11.7 [10.8, 12.5] days (Fig. 2a). The reported time taken to reach the maximum treatment effect was highly variable, with 16% reporting reaching peak effect within 3 weeks, 27% reporting reaching peak effect within 3–5 weeks and 20% reporting it takes longer than 5 weeks to reach peak effect. The remaining 36% of respondents were unable to describe the time to peak effect (answered “don’t know”). Of those who answered, the mean [95% CI] time to peak effect was 4.5 [4.2, 4.9] weeks.

### Experiences of motor symptom re-emergence

**Fig. 3:**
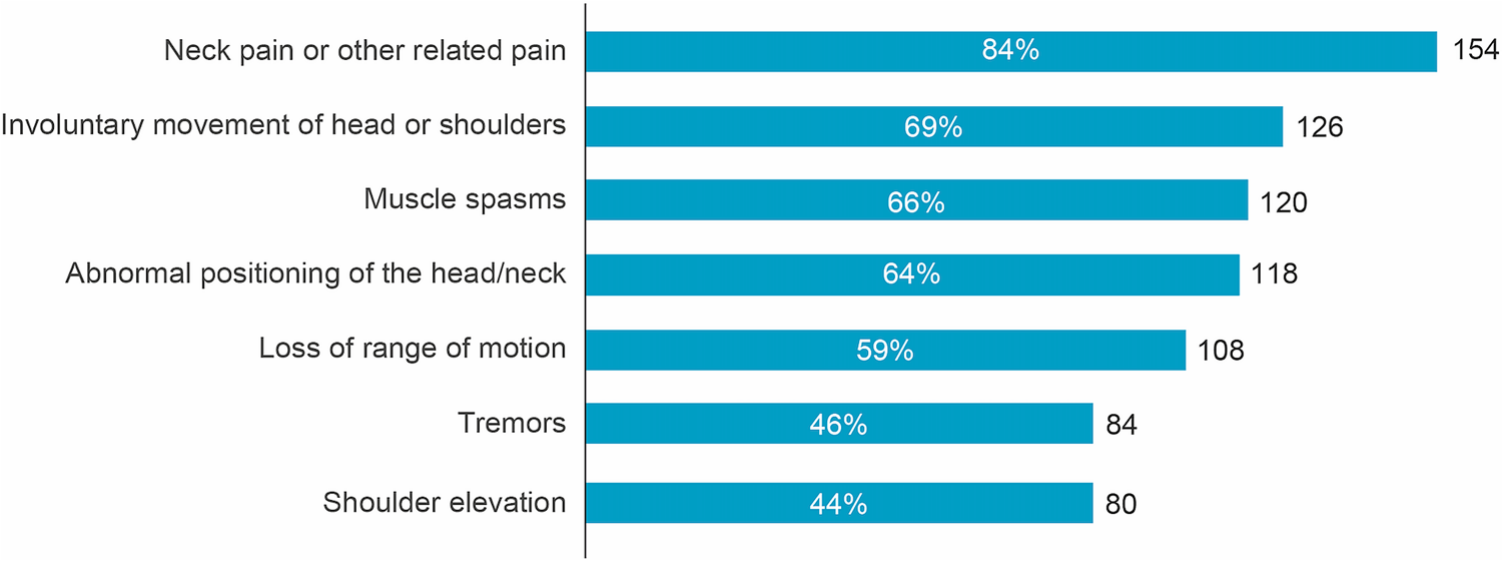
Re-emergent symptoms. Question: Select the [pre-existing symptoms] which reappear between two sessions of BoNT-A injections

Symptom re-emergence between injections was common, with 88% of respondents saying they noticed their pre-existing symptoms reappearing between injection sessions (Fig. 3). The mean [95% CI] time to re-emergence of pre-existing symptoms was 73.6 [69.7, 77.4] days, with 4% reporting symptom re-emergence within a month, 24% reporting within 1–2 months, 38% reporting 2–3 months and 16% reporting symptom re-emergence only after > 3 months. Overall, 33 patients (18%) could not define the time to re-emergence of pre-existing symptoms. Respondents receiving BoNT-A and concomitant oral medications experienced symptom re-emergence more frequently than those who only received BoNT-A injections and those who also received physiotherapy (92% vs. 85% and 77% of patients in each treatment group respectively).

On average, patients experienced 4.3 symptoms between two sessions of BoNT-A injections. Respondents were asked to rate the intensity of their symptoms at BoNT-A peak effect, at waning of effect and one day prior to their next injection. Treatment was not reported to completely abolish symptoms, even at peak effect. Figure 2b shows that respondents rated their symptom severity as low at the peak of BoNT-A treatment effects (mean scores 2.6–3.1/10), increasing as the effects of treatment start wearing off (5.4–5.8/10) and as high one day before the next session (7.1–8.0/10). The impact of CD recurring symptoms on quality of life followed the same ‘rollercoaster’ pattern (Fig. 2c): rated as lowest at the peak of treatment effects (2.1–2.7/10), with increasing impact as the effects of treatment waned (4.4–5.1/10) until the next injection session (6.3–7.0/10).

**Fig. 4:**
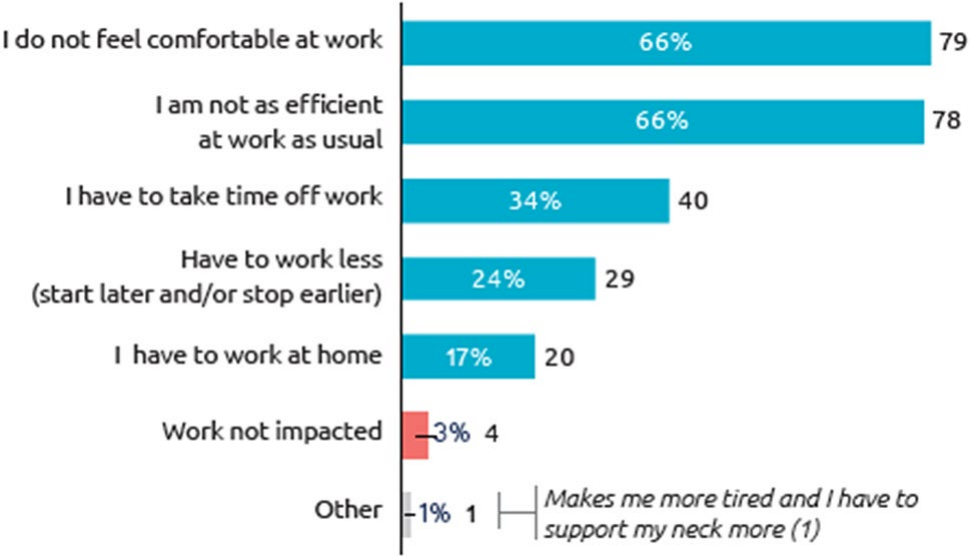
Impact of symptom re-emergence on professional life (working respondents). Question: How does the reappearance of your cervical dystonia pre-existing symptoms between two sessions of BoNT-A injections affect your work? (*N* = 119 working respondents). *BoNT-A* botulinum neurotoxin type A

When patients who were working (*n* = 119) were specifically asked about the impact of the reappearance of pre-existing CD symptoms between injection sessions on work, the vast majority (97%) reported some level of impact on their professional life; 66% said they do not feel comfortable at work and 66% said they are not as efficient at work as usual (Fig. 4).

### Physician–patient communication about symptom re-emergence

Most respondents (78%) said they had discussed the potential for symptom re-emergence between injections with their doctor; 11% said they had not discussed this possibility and 11% could not remember if this had been discussed or not. When asked whether they report symptom re-emergence to their physician, 34% (*n* = 54/160) said they inform their physician at the next appointment, and 24% (*n* = 39/160) said they report their symptom re-emergence immediately regardless of the intensity of symptoms. Overall, 14% of respondents (*n* = 22 of 160 patients experiencing symptom re-emergence) said they do not inform their doctor, and 28% (*n* = 45/160) said they only report when their symptoms are severe (18% immediately and 10% at the next appointment). Of the 22 respondents who said they do not report their symptom re-emergence, most (*n* = 15, 68%) said they “did not think their doctor could do anything about it”.

Following a report of symptom re-emergence, the most common management approaches were dose increase (54%), introduction of additional treatment (35%) and a reduction in the treatment interval (23%). A further 19% were told to wait for the next injection and 6% switched brands. Finally, when asked what improvements with BoNT-A treatment they wanted to avoid symptom re-emergence between sessions, most respondents who suffer symptom re-emergence (*n* = 130/183, 71%) said they would like a longer lasting BoNT-A treatment, while few considered shorter intervals (15%), higher doses (4%) or more muscles injected (4%).

## Discussion

To our knowledge, this is one of the first surveys to provide in depth evaluation of how patients living with CD experience the therapeutic effects of BoNT-A treatment and the quality of life impacts of symptom re-emergence between injection sessions. Similar to that reported by a previous survey [16], we confirmed peak BoNT-A therapeutic effects at 4.5 weeks, but with a generally predictable symptom re-emergence after about 10.5 weeks. We extend these observations by evaluating the prevalence and severity of symptom re-emergence in between injection sessions and show the waning of BoNT-A effect has important impact on patient daily activities and quality of life.

Although clinical studies have shown significant reductions (versus baseline and placebo) in Toronto Western Spasmodic Rating Scale (TWSTRS) scores as early as 1 week [11], our findings indicate that most patients only appreciate the benefits of treatment after at least 9 days. This time-lag to onset is longer than the 3.8 days reported by Sethi et al. [16] and may reflect differences in how patients were asked to estimate onset of effect. For example, within our survey, patients were asked to consider the impacts of CD considering motor (e.g., muscle spasms, abnormal positioning of the head/neck etc.) and non-motor aspects (e.g., fatigue, feeling sad or depressed etc.), and this may have influenced how they answered the question of when they experience the first effects of the treatment. Survey findings also showed considerable variation in the reported time to peak effects. However, taken on average, patient perception matched the clinical data (based on motor symptoms) which suggests that it takes 4–6 weeks for BoNT-A treatment to reach peak efficacy [10, 11].

The majority of respondents (90%) reported the reappearance of pre-existing symptoms between 2 injections. Among them, waning of effects were reported to start occurring about 10–11 weeks after the injection. This is in line with the time to waning of effect as reported for incobotulinumtoxinA and onabotulinumtoxinA (10.0 and 9.9 weeks, respectively) by Benecke et al. [17], but is shorter than the ‘treatment effect’ or subjective ratings of efficacy reported for other clinical studies [18]. This is likely because (1) most clinical studies assess patients at predefined time points (e.g., 8 and 12 weeks) and do not capture what happens in between and (2) the duration of treatment effect is typically calculated as the time taken to reach 80% of the baseline score [11, 17, 18]. Most clinical studies have not prospectively looked at time to symptom re-emergence/waning of effect. A recent review of the abobotulinumtoxinA clinical literature approached this question by reviewing the time to retreatment (i.e., length of injection intervals when patients were retreated according to investigator judgement) as an alternative metric and reported that in CD clinical trials, 72.6–81.5% of abobotulinumtoxinA patients did not require retreatment before 16 weeks [19].

In accordance with the time course of BoNT-A effects, the intensity of symptoms and their subsequent impact on quality of life ‘fluctuated’ widely at different points of treatment. It is noteworthy that patients reported experiencing a relatively low level of CD symptom intensity (scoring between 2 and 3 out of 10), even at peak BoNT-A effect. Symptoms were generally reported as being of moderate intensity when respondents reported they noticed symptom re-emergence and were moderate to severe one day before the next injection. A limitation of the survey is that we don’t know if the respondents equated a score of 10 ‘very strong symptoms’ with their worst severity experienced. This may have affected the results since a recent study suggests that patients don’t fully return to baseline disease severity between injections (as assessed by TWSTRS scores) [15].

The impact of recurring symptoms on quality of life followed the same pattern across all domains evaluated. CD symptoms similarly affected the respondents ability to work, have social interactions, drive, perform daily tasks and sleep. Even if most working-aged patients were still employed (73%), they often had to reduce their time spent at work because of their cervical dystonia: 30% of the patients have a part-time job and 19% do not work because of their cervical dystonia. Our data lend support to the role of effective treatment in maintaining employment status for people living with CD. In one study, Skogseid et al. reported that the employment rate for patients with CD fell from 84% at the onset of CD to 47% before initiation of BoNT-A treatment. With long-term BoNT-A treatment, 72% of those who worked at the initiation of treatment stayed employed, and 67% of those on sick leave returned to work [3]. However, the impact on those respondents who work was striking, 97% of working patients reported that their work was affected by returning symptoms; two thirds said they feel uncomfortable at work and the same proportion reported a loss of efficiency.

Our survey findings highlight the importance of the therapeutic partnership between physician and patient. Whilst the majority of respondents were satisfied with their injection scheduling, about 20% said their injection schedule was not well adapted to their needs and this would be something to discuss with their treating physician. The impact of healthcare access was also clear; most respondents (71%) whose injection sessions depend on doctor availability were unhappy with their schedule. While it is likely that differences in health system organisation in those being treated in UK and Italy (universal national health service), Germany and France (mixed systems with mutual insurers), and US (having mainly a private structure) play into perceptions of the therapeutic alliance, we did not observe any obvious country differences. Survey findings highlight that the effects of BoNT-A are not immediate, take time to reach their full potency and frequently do not last throughout the time interval between two sessions. Given the high expectations many patients have of BoNT-A therapy [20], it is important that they understand BoNT-A therapy is not a cure and the probable time course of symptom relief. It is also important that patients report back to their physician on their experiences of their treatment. In this survey, 14% of patients did not inform their doctor of the reappearance of their symptoms between injection sessions, mostly because they believe their doctor cannot help them. Physicians can only adjust the treatment regimen if they have adequate information to hand, and this will often depend on the patient being able to communicate how and when they experience their symptoms. To this end, it may be helpful to develop simple patient tools explaining what to expect from BoNT-A treatment and allowing them to record their experience. Our survey shows that most patients are able to clearly describe the waning effect. Such tools have been developed in other movement disorders such as Parkinson’s disease and have been reported to improve the physician–patient dialogue [21, 22].

When respondents were asked what they would like for their BoNT-A therapy, most indicated they would prefer a regimen with longer injection intervals highlighting their need for long lasting symptom relief between BoNT-A injections. This is in direct contrast to a prior survey where patients indicated they would prefer shorter intervals to match their duration of efficacy [16]. The reasons for this discrepancy are unclear but may, for example, reflect the way the questions were framed. On the other hand, our data lend support the findings of the recent INTEREST IN CD2 study, where longer injection intervals were a significant predictor of satisfaction with symptom control both at peak effect and end of cycle [15]. There is a clear BoNT-A dose dependency for duration of effect, and in cases of shorter than desired intervals, clinicians can consider several ways of improving the regimen such as increasing the dose of BoNT-A delivered to the affected muscle. Another intriguing finding from our survey are the differing frequencies of symptom re-emergence according to use of concomitant therapies—only 26% of patients included were treated with BoNT-A solely, while the remaining patients were treated with combination of BoNT-A, physiotherapy, and/or oral medications. While this makes it difficult to disentangle the isolated effect of BoNT-A injections, it represents the real life situation, and a high proportion of respondents in all subgroups experienced symptom re-emergence. Whereas the higher frequency of symptom re-emergence in respondents receiving concomitant oral therapies may reflect a worse disease severity than those managed with BoNT-A alone, the lower frequency of symptom re-emergence in patients receiving concomitant physiotherapy (and given that < 20% of patients receive physiotherapy) merits attention in future work. Although the quality of evidence for physiotherapy following BoNT-A injections in CD is generally low [23], small studies have reported a therapeutic benefit of the combination [24, 25].

Limitations of this study are those inherent to patient surveys, which are based on the respondent’s own understanding of their condition and are not cross checked with clinical information. There was no verification of the CD diagnosis other than self-report, and we did not consider the type of CD the respondent has or their treatment setting (e.g., movement disorders specialist vs. general neurologist) and how this may affect the BoNT-A experience. Another limitation is the recruitment method in which only those respondents with access and skills in Internet use would be likely to respond to the survey. The Carenity platform likely engages the most motivated of patients and we cannot be sure if the respondents represent a more severely affected or treatment refractory CD population. Likewise, we do not know how many people were directed to the website from the CD patient societies, and therefore cannot fully comment on this recruitment bias. Finally, although we captured the overall prevalence of non-motor problems such as fatigue, depression, sleep and sexual dysfunction, our exploration of symptom re-emergence following BoNT-A injections was more focused on motor symptoms. The presence of non-motor symptoms significantly worsens quality of life in CD [26], and future work should consider whether treatment of the core motor symptoms indirectly alleviates this huge, largely unrecognised burden.

In summary, the results of this survey highlight the burden of CD symptoms, even in patients undergoing regular treatment. The survey found that patients living with CD can expect certain time profile of BoNT-A effects, namely a short wait to onset, a time lag to peak effect and a gradual decline in efficacy thereafter. Symptom re-emergence is common and has significant impact on daily activities and quality of life. Greater patient—and physician—awareness of this therapeutic profile should lead to better informed therapeutic discussions and planning.

## Electronic supplementary material

Below is the link to the electronic supplementary material.

Supplementary file1 (PDF 297 kb)

## Data Availability

Where data can be anonymised, Ipsen will share all individual participant data that underlie the results reported in this article with qualified researchers who provide a valid research question. Proposals should be submitted to DataSharing@Ipsen.com and will be assessed by a scientific review board. Data are available beginning 6 months and ending 5 years after publication; after this time, only raw data may be available.
