# Correction to: Patient perspectives on the therapeutic profile of botulinum neurotoxin type A in cervical dystonia

**DOI:** 10.1007/s00415-020-10255-1

**Published:** 2020-10-26

**Authors:** Cynthia Comella, Joaquim J. Ferreira, Emilie Pain, Marion Azoulai, Savary Om

**Affiliations:** 1 Department of Neurology, Rush University Medical Center, 1725 West Harrison St. Suite 755, Chicago, IL 60612 USA; 2 Instituto de Medicina Molecular, Faculdade de Medicina, Universidade de Lisboa, Lisbon, Portugal; 3 CNS, Campus Neurológico Sénior, Torres Vedras, Portugal; 4 Carenity, Paris, France; 5 Global Medical Affairs, Ipsen Pharma, Boulogne-Billancourt, France

## Correction to: Journal of Neurology 10.1007/s00415-020-10217-7

The article Patient perspectives on the therapeutic profile of botulinum neurotoxin type A in cervical dystonia, written by Cynthia Comella, Joaquim J. Ferreira, Emilie Pain, Marion Azoulai and Savary Om, was originally published electronically on the publisher’s internet portal on 16 September 2020 without open access. With the author(s)’ decision to opt for Open Choice the copyright of the article changed on 25 September 2020 to © The Authors 2020 and this article is licensed under a Creative Commons Attribution 4.0 International License, which permits use, sharing, adaptation, distribution and reproduction in any medium or format, as long as you give appropriate credit to the original author(s) and the source, provide a link to the Creative Commons licence, and indicate if changes were made. The images or other third party material in this article are included in the article’s Creative Commons licence, unless indicated otherwise in a credit line to the material. If material is not included in the article’s Creative Commons licence and your intended use is not permitted by statutory regulation or exceeds the permitted use, you will need to obtain permission directly from the copyright holder. To view a copy of this licence, visit https://creativecommons.org/licenses/by/4.0/

The original article has been corrected.

