# Supplementary material for: Patient perspectives on the therapeutic profile of botulinum neurotoxin type A in cervical dystonia
Source: J Neurol. 2020 Sep 16;268(3):903–12. doi: 10.1007/s00415-020-10217-7 (PMC7914227; doi:10.1007/s00415-020-10217-7)
Supplement: Supplementary file 1 — Supplementary file1 (PDF 297 kb) [file 415_2020_10217_MOESM1_ESM.pdf]

Understanding the waning of BoNT-A effects and its impact on the quality of life of patients living with cervical dystonia

Description message:

Hello,

We invite you to participate in an international survey designed **to better understand patients' experience with Botulinum Toxin A injections**. These injections are given to improve symptoms related with cervical dystonia.

As a patient, your feedback about symptoms and treatments will be useful to better understand your needs and how we can improve treatments.

The data collected through this study will only be **shared anonymously and in aggregate form**, which means that it will not be possible to identify the participants.

These aggregate results will be shared with Ipsen, a pharmaceutical company that manufactures a treatment for spasticity and cervical dystonia.

This study will be also the subject of **scientific publications** that will be redacted in collaboration with an **international team of 4 medical experts.**

According to the current legislation, you have the right to access, rectify and oppose to the treatment of your data.

To learn more about the way your data will be treated and about the terms of exercise of your rights, please read our Information and Consent notice.

Your participation in the survey is voluntary. By clicking on the "Start" button, you confirm:
1. your willingness to participate in the study; and
2. that you give your explicit consent to have your personal data collected and processed in the way described in our Information and Consent Notice.

You may withdraw your consent at any time without providing a reason, without affecting the lawfulness of the processing carried out prior to the withdrawal of your consent.

Thank you in advance for your contribution,

The Carenity Team

During the questionnaire, you will be asked **the name of *the last* *Botulinum Toxin A injections you received***. *If you do not remember, or are not sure about the name, please refer to your doctor or your medical centre before starting the survey. This information can also appear on a medical report.*

This questionnaire should take you 10 to 15 minutes to complete. Once you have finished answering a question, you can click on the "Next" button to go to the next question. Please note that you will not be able to move back to the previous question after that.

Thank you for your participation.

A. Respondent's profile

1. You are a:

(Single answer)

- ◻ Man
- ◻ Woman

2. Your year of birth:

(yyyy) **[SCREEN OUT if age<18 y/o]**

3. Your country of residence:

(Single answer)

- ◻ France
- ◻ Germany
- ◻ Italy
- ◻ Spain
- ◻ UK (England, Wales, Scotland, Northern Ireland)
- ◻ USA
- ◻ Other *[Specify]* **[SCREEN OUT]**

4. Your profile:

(Single answer)

- ◻ Patient with a cervical dystonia
- ◻ Not suffering with cervical dystonia
- ◻ **[SCREEN OUT]**

5. **Please tell us about your employment status.**
**Full-time: more than 30 hours per week*
***Part-time: less than 30 hours per week*
(Single answer)
□ I work full-time*
□ I work part-time** because of my condition
□ I work part-time** but it is not due to my condition
□ I do not work because of my condition
□ I do not work but it is not due to my condition (retired, …)
□ I am a full time student
□ Other *[Specify]*

6. **When did the first symptoms of your cervical dystonia appear?**
(yyyy)
□ I do not remember

7. **When were you diagnosed with cervical dystonia?**
(yyyy)
□ I do not remember

8. **During the past 12 months, at the worst time, which of the following symptoms have you experienced as a consequence of your cervical dystonia?**
(Several possible answers)
□ Muscle spasms
□ Involuntary movement of head or shoulders
□ Neck pain or other related pain
□ Abnormal positioning of the head/neck
□ Loss of range of motion (inability to move the head easily)
□ Tremors
□ Shoulder elevation
□ Other *[Specify]*

9. **During the past 12 months, at the worst time, which of the following situations have you experienced as a consequence of your cervical dystonia?**
(Several possible answers, randomized)
□ Loss of self-confidence due to stigma of visible head/neck dystonia
□ Fatigue or lack of energy which limits daytime activities
□ Any walking difficulty or balance problem
□ Difficulties falling or staying asleep
□ Feeling not refreshed after an overnight sleep
□ Feeling sad or depressed
□ Feeling nervous, worried or frightened for no apparent reason
□ Problems with or less interested in sexual activities
□ Experience of unpleasant sensations such as numbness, tingling or pins and needles
□ Pain, not explained by other conditions
□ Experience of light-headedness or dizziness
□ Flat moods without the normal ‘highs’ and ‘lows’
□ Dystonia is affecting vision
□ Any speech problems

- ☐ Difficulties while eating such as chewing or swallowing
- ☐ Difficulties at work
- ☐ Other *[Specify]*
- ☐ None

[In the following questions, we will ask the respondents about their current and past treatments. Respondents who have not received Botulinum Toxin A injections will be screened out (Q10 and Q11).]

10. Which treatments are you currently receiving for your cervical dystonia?
(Several possible answers)
- ☐ Oral (tablet or liquid) medication (muscle relaxant, oral baclofen…)
- ☐ Botulinum toxin A injections (injections into the muscle: Botox®, Dysport®, Xeomin®, brand unknown)
- ☐ Botulinum toxin B injections (injections into the muscle: MyoBloc®, NeuroBloc®, brand unknown)
- ☐ Physiotherapy at home
- ☐ Physiotherapy at hospital/clinic
- ☐ Surgery/deep brain stimulation
- ☐ Psychological support/counselling support
- ☐ Traditional/complementary medicine (e.g. Chinese medicine, osteopathy, homeopathy, etc)
- ☐ Other *[Specify]*
- ☐ I do not take any specific treatment for my cervical dystonia

11. In the past, which treatments did you receive for your cervical dystonia?
(Several possible answers)
- ☐ Oral (tablet or liquid) medication (muscle relaxant, oral baclofen…)
- ☐ Botulinum toxin A injections (injections into the muscle: Botox®, Dysport®, Xeomin®, brand unknown)
- ☐ Botulinum toxin B injections (injections into the muscle: MyoBloc®, NeuroBloc®, brand unknown)
- ☐ Physiotherapy at home
- ☐ Physiotherapy at hospital/clinic
- ☐ Surgery/deep brain stimulation
- ☐ Psychological support/counselling support
- ☐ Traditional/complementary medicine (e.g. Chinese medicine, osteopathy, homeopathy, etc)
- ☐ Other *[Specify]*
- ☐ I did not take any specific treatment for my cervical dystonia in the past

[Respondents who have not received Botulinum Toxin A injections will be screened out (Q10 and Q11).]

[For patients who received BoNT-A injections in the past only]

12. Did you stop receiving Botulinum Toxin A injections more than one year ago?
(Single answer)
- ☐ Yes **[SCREEN OUT]**
- ☐ No

B. Experience with BoNT-A injections

[For patients who received BoNT-A injections in the past only]

INSERT:
For the next part of the questionnaire, we are going to ask you questions about your Botulinum Toxin A injections. **Please answer for the period of time you were receiving Botulinum Toxin A injections**.

13. What is the name of the Botulinum Toxin A injections you last received?
If you don't remember the name of the last Botulinum Toxin A injections received, please refer to your doctor or your medical centre. This information can also appear on a medical report.
(Single answer)
- □ Botox®
- □ Dysport®
- □ Xeomin®
- □ I do not know

14. When did you start receiving Botulinum Toxin A injections?
(yyyy)
- □ I do not remember

15. In total, since Botulinum Toxin A treatment was started, how many times did you receive Botulinum Toxin A injections for your cervical dystonia?
(Single answer)
- □ 1 session of injections **[SCREEN OUT]**
- □ 2 sessions of injections
- □ 3 sessions of injections
- □ 4 sessions of injections **or more**

16. On average, how many times per year do you receive Botulinum Toxin A injections?
(Minimum=1, maximum=6)
(Numeric)
- □ I do not know

17. When did you receive your last session of Botulinum Toxin A injections for your cervical dystonia?
(Single answer)
- □ Less than 1 month ago
- □ 1 month to less than 2 months ago
- □ 2 months to less than 3 months ago
- □ 3 months to less than 4 months ago
- □ 4 months to less than 6 months ago
- □ 6 months ago or more
- □ I do not remember

18. What was the time between your two last sessions of Botulinum Toxin A injections for your cervical dystonia?

(Single answer)

- □ Less than 3 months
- □ 3 months to less than 4 months
- □ 4 months to less than 6 months
- □ 6 months or more
- □ I do not remember

19. Please select the answer which best describes how your sessions of Botulinum Toxin A injections are scheduled.

(Single answer)

- □ The interval between two sessions of injections is always the same.
- □ The interval between two sessions of injections is not regular, the sessions are arranged when needed depending on my symptoms.
- □ The interval between two sessions of injections is not regular, the sessions are arranged depending on the availability of the doctor/hospital.
- □ Other [*Specify]*

19bis. What do you think about the way your sessions of Botulinum Toxin A are scheduled?

(Single answer)

- □ It's well adapted to my situation.
- □ I would like the sessions to be arranged differently.
- □ Other [*Specify]*

20. On average, how many days or weeks after your Botulinum Toxin A injections do you experience:

(Numeric, minimum=0)

[The first effect of the treatment on your cervical dystonia] (in days or weeks) □ I do not know

[The maximum effects of the treatment on your cervical dystonia] (in day or weeks) □ I do not know

C. Experience with the waning of BoNT-A effects

21. During your first sessions of Botulinum Toxin A injections, did your doctor inform you that symptoms could return between two sessions of injections?

(Single answer)

□ Yes

□ No

□ I don't remember

22. In general, do your cervical dystonia pre-existing symptoms reappear between 2 sessions of Botulinum Toxin A injections?

(Single answer)

□ Yes

□ No **[Respondents who checked "no" and are still receiving BoNT-A treatment will be sent to the end of the questionnaire]**

23. In general, how long after your Botulinum Toxin A injections do your cervical dystonia pre-existing symptoms begin to reappear?

(Numeric, minimum=0)

(in days or weeks) □ I do not know

24. Among the following pre-existing symptoms, could you select the ones which reappear between two sessions of Botulinum Toxin A injections in their order of reappearance?

The first symptom you select is the one which reappears first and so on. If a symptom does not appear between two Botulinum Toxin A treatments, please do not select it. If you want to change the order, or remove a symptom from the ranking, you can unselect it by clicking on it again.
1: this symptom reappears first, 2: this symptom is the second to reappear…
(Ranking question, minimum 1 item)

□ Muscle spasms

□ Involuntary movement of head or shoulders

□ Neck pain or other related pain

□ Abnormal positioning of the head/neck

□ Loss of range of motion (inability to move the head easily)

□ Tremors

□ Shoulder elevation

[Question 25 will be asked for <u>each symptom selected</u> at question 24]

25. At these 3 different points of treatment with Botulinum Toxin A, <u>how would you rate the intensity of your</u> [SYMPTOMS Q24] as a consequence of your cervical dystonia?

(1 answer per line, 0 = No symptoms, 10 = Very strong symptoms)
For each aspect, drag the slider to the desired position: left end = no symptoms, right end = very strong symptoms.

When the effects of the injections are at their maximum (peak effect)	No symptoms -------------O------------ Very strong symptoms
When the pre-existing symptoms reappear/ the effects of the injections start wearing off	No symptoms -------------O------------ Very strong symptoms
The day before receiving new injections	No symptoms -------------O------------ Very strong symptoms

D. Impact of the waning of BoNT-A effects on the patient's quality of life and expectations

[Q26 will be asked for patients currently working only]

26. How does the <u>reappearance of your cervical dystonia pre-existing symptoms</u> between two sessions of Botulinum Toxin A injections affect your work?

(Several possible answers)

- ◻ I have to take time off work
- ◻ I do not feel comfortable at work
- ◻ I have to work less (start later and/or stop earlier than I would otherwise, …)
- ◻ I have to work at home (homeworking)
- ◻ I am not as efficient at work as I usually am
- ◻ Other *[Specify]*
- ◻ It does not affect my work

[Q27 will be asked for the five following items:]

- **ability to perform daily tasks (e.g. grooming, dressing…)**
- **ability to work**
- **ability to drive**
- **ability to have social interactions**
- **sleep disturbance**

27. At these 3 different points of treatment with Botulinum Toxin A, how would you rate the impact of your cervical dystonia on your [QUALITY_OF_LIFE_ITEM]?

(1 answer per line, 0 = No impact, 10 = Very strong impact)

When the effects of the injections are at their maximum (peak effect)	No impact -------------O------------ Very strong impact
When the pre-existing symptoms reappear/ the effects of the injections start wearing off	No impact -------------O------------ Very strong impact
The day before receiving new injections	No impact -------------O------------ Very strong impact

28. When the symptoms of your cervical dystonia reappear between two sessions of Botulinum Toxin A injections, do you inform your doctor about it?
(Several possible answers)
- ◻ Yes, I inform my doctor immediately regardless of the intensity of my symptoms
- ◻ Yes, I inform my doctor immediately but only when my symptoms are severe
- ◻ Yes, I inform my doctor at the next appointment regardless of the intensity of my symptoms
- ◻ Yes, I inform my doctor at the next appointment but only when my symptoms are severe
- ◻ No **> Q30**

[Q29 is only asked for patients who have informed their doctor about the reappearance of their pre-existing symptoms]

29. How did your doctor react when you informed him/her about the earlier reappearance of your cervical dystonia symptoms?
(Several possible answers)
- ◻ My doctor prescribed an additional treatment (e.g. oral treatment/physiotherapy) to combat the symptoms
- ◻ My doctor increased the dosage of the Botulinum Toxin A injection used in the next treatment session
- ◻ My doctor switched my Botulinum Toxin A to another brand
- ◻ My doctor asked me to come earlier for the next Botulinum Toxin A injection
- ◻ My doctor told me I have to wait for the next injection
- ◻ Other *[Specify]*
- ◻ I do not know

[Q30 is only asked for patients who have not informed their doctor about the reappearance of their pre-existing symptoms]

30. Why did you not inform your doctor about the earlier reappearance of your cervical dystonia symptoms?
(Several possible answers)
- ◻ I did not think my doctor could do anything about it
- ◻ I did not want to disturb my doctor
- ◻ I did not have time to inform my doctor
- ◻ My doctor did not have the time
- ◻ I was worried that my doctor would prescribe an additional treatment (e.g. oral treatment/physiotherapy)
- ◻ I did not want to be prescribed Botulinum Toxin A injections more frequently
- ◻ I did not want to have more muscles injected during the same injection session
- ◻ I did not want to be prescribed a different Botulinum Toxin treatment
- ◻ I was worried that my doctor would not believe me
- ◻ Other *[Specify]*
- ◻ I do not know

31. What improvements with your Botulinum Toxin A treatment do you want in order to avoid reappearance of symptoms between Botulinum Toxin A injection sessions? Please select the most important one:
(Single answer)
- ◻ Have a treatment with benefits lasting longer
- ◻ Have more muscles injected
- ◻ Have higher doses injected
- ◻ The possibility to have more frequent injections
- ◻ Other *[Specify]*
- ◻ None

E. Reasons for stopping BoNT-A injections

[For patients who have stopped Botulinum Toxin A injections only]

32. Why did you stop receiving Botulinum Toxin A injections for your cervical dystonia?
(Several possible answers)

- ☐ I had side effect(s) [*Specify*]
- ☐ The treatment did not work well enough
- ☐ My doctor told me to stop the injections
- ☐ The treatment was too expensive
- ☐ Logistics constraints (travel to hospital, time spent on injections…) were too inconvenient
- ☐ I wanted to take less medication
- ☐ Injections were too painful
- ☐ Another treatment was proposed as an alternative to Botulinum Toxin injections (e.g. surgery)
- ☐ I did not need it anymore
- ☐ Other *[Specify]*
- ☐ I don’t remember
